# Appropriate controls for digital therapeutic clinical trials: A narrative review of control conditions in clinical trials of digital therapeutics (DTx) deploying psychosocial, cognitive, or behavioral content

**DOI:** 10.3389/fdgth.2022.823977

**Published:** 2022-08-18

**Authors:** Jacqueline Lutz, Emanuela Offidani, Laura Taraboanta, Shaheen E. Lakhan, Timothy R. Campellone

**Affiliations:** ^1^ Medical Office, Click Therapeutics Inc., New York, NY, United States; ^2^ Clinical Epidemiology Research in Medicine, Weill Cornell Medicine, New York, United States; ^3^ School of Neuroscience, Virginia Tech, Blacksburg, VA, United States

**Keywords:** mHealth, psychology, digital clinical trials, digital health, control conditions, placebo control, software as a medical device, sham

## Abstract

Digital therapeutics (DTx) are software programs that treat a disease or condition. Increasingly, DTx are part of medical care, and in the US healthcare system they are regulated by the FDA as Software as a Medical Device (SaMD). Randomized controlled trials (RCT) remain a key evidence generation step for most DTx. However, developing a unified approach to the design of appropriate control conditions has been a challenge for two main reasons: (1) inheriting control condition definitions from pharmacotherapy and medical device RCT that may not directly apply, and (2) challenges in establishing control conditions for psychosocial interventions that build the core of many DTx. In our critical review we summarize different approaches to control conditions and patient blinding in RCT evaluating DTx with psychosocial, cognitive or behavioral content. We identify control condition choices, ranging from very minimal digital controls to more complex and stringent digital applications that contain aspects of “fake” therapy, general wellness content or games. Our review of RCTs reveals room for improvement in describing and naming control conditions more consistently. We further discuss challenges in defining placebo controls for DTx and ways in which control choices may have a therapeutic effect. While no one-size-fits-all control conditions and study designs will apply to all DTx, we propose points to consider for defining appropriate digital control conditions. At the same time, given the rapid iterative development and optimization of DTx, treatments with low risk profile may be evaluated with minimal digital controls followed by extensive real-world effectiveness trials.

## Introduction

Digital therapeutics (DTx), a class of software-based (internet- and/or app based) technologies, aim to directly prevent, manage or treat health conditions (1). DTx often translate face-to-face treatment modalities, such as cognitive behavioral therapy (CBT), into mobile application or web-based interventions. Self-guided internet-based cognitive behavioral therapy programs (iCBT) have been studied extensively (2), but many DTx technologies contain treatment principles beyond iCBT, such as cognitive training components or symptom tracking/medication adherence. DTx have the potential to revolutionize modern medicine by improving access to evidence-based, personalized treatments that may for some indications even evolve into first-line therapy. As such, DTx - whether as standalone therapy or adjunctive to medications, devices, or other therapies - have shown promise for a broad spectrum of medical conditions ranging from mental health and behavioral health conditions (3) (e.g., depression (4), insomnia (5), post-traumatic stress disorder (PTSD) (6), attention deficit hyperactivity disorder (ADHD) (7), and substance use disorders (8)) to managing conditions such as diabetes (9), irritable bowel syndrome (10) or post-stroke aphasia (11).

At the same time, the relatively novel field of DTx is still in search of common standards for how to generate evidence necessary to support medical claims and regulatory clearance (12). Here, we will discuss the concept of control conditions in randomized controlled trials (RCT) in the context of pharmacological and psychotherapeutic trials, and within the FDA regulatory framework for Software as a Medical Device (SaMD) that DTx fall under. We will then review and discuss typical control conditions that DTx companies have used in their RCTs to date.

### Randomized controlled trials and control conditions

RCTs are considered the “gold standard” to evaluate therapeutic interventions (13). In RCTs, participants are either allocated to the active treatment or a control condition. Different control conditions can be distinguished based on their stringency, what biases they control for. The control condition a treatment is compared to is therefore a crucial choice as it directly impacts the measurable treatment effect, with less stringent control conditions leading to larger effect sizes.

In the early clinical development phase, control conditions are often less stringent. For example, waitlist or no intervention control groups have been used. These will control for effects related to the natural progression of the illness, regression to the mean effects (14), effects from regular social interaction with study trial personnel, or effects from other study procedures such as being assessed repeatedly or simply from feeling observed during a trial (i.e., Hawthorne effect) (15, 16). However, studies employing these control groups are not blinded, and thus do not control for the expectation of benefit.

 In later clinical development phases, more stringent placebo control conditions are typically used. In pharmacological trials, placebo pills look exactly like the active treatment, but do not contain any active ingredients. Participants are told during the informed consent process that they will be randomized to either the treatment or placebo condition, and if blinding to the assignment was successful, the placebo pill condition controls for any bias due to any non-specific mechanisms beyond the physiological effects that the active drug ingredients have on the individual (17). Thus, the placebo pill will also control for expectation of change during a trial (see Table 1 for definitions).

**Table 1:** Definitions.

**Placebo effect**	Distinctive psychobiological phenomenon based on expectation of benefit or effects related to practitioner-patient encounter (14, 41).
**Placebo response**	Response to receiving placebo in clinical trials. This includes the placebo effect but also includes noise related to bias in reporting, regression to mean, natural disease progression and possibly Hawthorne effects (41).
**Waitlist control**	A control condition in which a group waits to receive the treatment later and is compared to a group that receives the treatment immediately. The two groups are not matched regarding their expectation of benefit, which may overestimate treatment effects.
	**Typically used in:** Early phase discovery and clinical development studies.
**Treatment as usual (TAU) control**	A control group that will receive standard of care. The level of care may vary per indication and level of TAU standardization between trials may vary and may be closer to an active control condition.
	**Typically used in:** This type of control is often used in pragmatic, real-world late phase trials.
**Placebo Control (“Sham”) (FDA)**	“Control group may be another device, simulated procedure or possibly a drug or biological product that is believed to have no therapeutic (or diagnostic) effect” (42).
	**Typically used in:** late phase (registration) studies.
**Active Intervention Control (FDA)**	“Control group provides another intervention (usually another device or surgery, but possibly a drug or biological product) that delivers a known effect” (42).
**Active control group in cognitive training/gaming studies**	Control group receives a similar therapy that does not specifically target the disorder or is shorter or less adaptive. Participants usually expect to receive a potential active treatment (no mentioning of sham). Active controls in this context control for structural aspects of the intervention and expectation of benefit but may not be fully inert (43).
**Attention matched control and Attention matched placebo control**	Terms proposed in a comparative efficacy literature. However, attention matched (placebo) controls may encompass a large variety of control choices (44).

Control conditions for psychological interventions are more challenging to design for a few reasons. First, while CBT and related psychosocial therapies are regarded as generally efficacious (18), the exact mechanisms that produce therapeutic effects in cognitive and behavioral therapies are not fully understood (19). This makes it challenging to define which treatment components should be included into the design of a control condition that is not intended to produce a therapeutic effect, similar to a placebo (sometimes referred to as attention control in psychotherapy research). Similarly, the relative contribution of factors shared across therapies, referred to as common factors (20) (e.g., therapeutic support, positive expectations of treatment benefit, hope, structure provided by the treatment (21)) is relatively large across different psychological interventions compared to specific treatment effects on disease symptoms (22, 23). In fact, a supportive therapist-client relationship would be considered part of the placebo control (often referred to as attention placebo control in this literature) in the context of a pharmacological trial, but the therapeutic alliance has been suggested as a common efficacy factor in psychotherapy^12^. Thus, a control condition which establishes structured and supportive therapeutic alliance already contains major efficacy factors of psychotherapy and will therefore not be inactive. On the flip side, trying to design placebo therapies with low or no active ingredients often result in therapies with low face-validity and the risk to unblind all or at least some participants to study assignment (22).

Because DTx often translate face-to-face treatment modalities, such as CBT, into mobile application or web-based interventions, they inherit the described challenges in defining appropriate controls of psychological interventions in general. In addition to disease specific therapeutic content, many DTx integrate disease management features (e.g., disease/symptom tracking, behavior tracking, goal setting and tracking, community support, disease related psychoeducation or general lifestyle and wellness content), which have been studied individually and shown positive effects for some indications (24–26). DTx may also deploy cognitive remediation and cognitive training principles to strengthen aspects of cognition, such as cognitive control or emotion regulation (27, 28). See also Table 1 in the **Supplementary Material** for a description of typical DTx treatment components. DTx that contain several interacting components as described above – i.e. disease specific therapeutic content, additional disease management tools, cognitive interventions and even coaching and text messages – often present complex interventions, and the particular challenge of studying complex interventions is well described and links back to a good understanding of the treatment mechanisms under investigation (29).

Some authors argue that designing a placebo control for DTx is easier compared to traditional face-to-face therapy since there is no human therapist interaction that could confound trial results (14). However, there may be digital placebo mechanisms beyond human interaction, whose impacts are not fully understood, such as beliefs about technology or the feeling of being connected to coaches or a therapist through using a digital health app (30). In addition, beyond typical placebo effects, there is currently a lack of understanding of efficacy related to the digital mode of delivery, such as the intervention structure, engagement support, or regular, focused time spent in the app (31). Most DTx recommend regular, often daily use (27, 32, 33), and deliver engaging experiences (e.g. gamification (27, 34) or social support (33)) to support adherence. However, such elements may share considerable overlap with disease specific therapeutic techniques. For example, the regular interaction with an engaging DTx may share elements with behavioral activation, a therapeutic activity with the goal of exposing patients to pleasurable activities that has been shown to be an effective treatment across a number of diseases (e.g. depression (35, 36), chronic pain, personality disorders, and schizophrenia (37)). Another example is the regular, scheduled interaction with a DTx, which overlaps with structuring the environment, a component of treatment for children with ADHD (38). Finally, if the DTx deploys game elements, additional therapeutic mechanisms could lie in distraction from negative affect or induction of positive affect through mastery and flow (39, 40). In summary, providing structure and creating positive engaging experiences and rewards may improve symptoms in addition to the specific therapeutic content. And while it is in the interest of patients to optimize and fully harness such mechanisms, this means that digital control conditions, which employ structurally equivalent protocols and engagement features may not be truly inert but have some therapeutic efficacy.

### Additional control considerations related to US/FDA regulations

DTx seeking FDA clearance fall into the category of SaMD and undergo testing and clinical validation like traditional medical devices. Several DTx have been authorized or cleared by the FDA as SaMD (e.g. reSET (8, 45), reSET-O (46), Somryst (5), EndeavorRx (27), Parallel (47)) and several digital health companies have communicated pursuing FDA clearance (e.g. Click Therapeutics (48), Kao Health (49), Posit Science (34), Wise Mind (50)).

The FDA provides guidance on suitable control conditions and blinding for evidence generation. The FDA describes a “sham”, the medical device equivalent of a placebo control in pharmacological trials, as being “an ineffective device (or simulated procedure or possibly a drug or biological product) used under conditions designed to resemble the conditions of use under investigation as far as possible” (42). In the case of SaMD, where treatment is based on software only, creating the equivalent of a placebo control poses particular challenges, a challenge even acknowledged by the FDA, which states that “it may be challenging to construct a placebo control that appears to function like the investigational device but delivers no therapy” (51). Specifically, it may be hard to keep appropriate face-validity to ensure patient blinding and comparable engagement with a placebo control while creating something that is “ineffective” (i.e., delivers “no therapy”). The FDA also outlines several other types of control groups, such as active controls (“an effective regimen of therapy may be used for comparison”. However, proving statistically that a treatment performs similar to a standard treatment (non-inferiority analysis) is challenging in practice because they rely themselves on strong historical placebo controlled RCT data to inform the non-inferiority margin and require larger sample sizes (52, 53). Taken together, the FDA guidelines applicable for SaMD follow typical medical device settings, which usually contain a hardware component, without concrete recommendations for control conditions in software only SaMD interventions. We expect additional guidance specific to SaMD may be issued by FDA and other regulatory bodies over time as best practices and standards emerge from the DTx industry, as the published literature evolves on the topic, and as further regulatory precedents are established through additional SaMD marketing clearances.

In summary, challenges of selecting appropriate control conditions for DTx, stem from the breadth of underlying treatment principles and mechanisms, sometimes interacting in a complex nature, known challenges in designing placebo controls for psychological treatments, and the fact that no physical device can aid in the blinding of participants. Here we review control condition choices in this nascent field of DTx and how DTx companies define appropriate controls for software based medical devices.

## Methods

In this narrative review, we explore control conditions in DTx RCTs deploying cognitive and/or behavioral therapeutic activities to manage or treat diseases. We reviewed the DTx Alliance product list (https://dtxalliance.org/understanding-dtx/product-library, accessed Jan 2022) to identify DTx that adhered to the DTx definition and core principles (eg. safety, efficacy, privacy, patient centricity). From this starting point, we focused on DTx which are purely software-based (internet or app) and do not contain additional physical devices for their functioning (e.g., wearables, inhalers, sensors), and where the primary activity was a cognitive or behavioral intervention. There were 10 products that fulfilled this criterion. From these, we explored phase 2 and 3 RCTs including those used to provide the evidence for FDA registration. In the case of several RCTs for a specific DTx, we focused on the most recent trial. To gain a broader picture of the fast-moving field, the authors added additional trials from DTx companies who planned and/or conducted phase 2 and 3 trials to support regulatory submission, based on their knowledge in the field of DTx and the companies press-releases (including a few planned, halted, or failed trials). For a full overview of reviewed DTx characteristics see Table 1, **Supplementary Material**).

## Results

### General summary

Fourteen RCTs were reviewed (Table 1 in the **Supplementary Material**). Sample sizes ranged from 80 to 1149. Most RCTs deployed 2 arms, but two studies ran 3 arms. Indications covered several DSM (54) categories (insomnia, schizophrenia, compulsive disorders, substance related disorders, depression, anxiety), neurodevelopmental disorders (ADHD) and physical disorders (diabetes, irritable bowel syndrome). A full overview of study characteristics is available in Table 1, **Supplementary Material**.

### Summary of control conditions

Several control strategies have been employed. We found about half of the trials used unblinded waitlist or treatment-as-usual (TAU) control groups. The other half deployed different forms of sham controls. Only one trial deployed an active comparator and only as part of a 3-arm RCT including a TAU control arm for the main comparison.

#### Waitlisted RCT

We identified three RCTs in Depression, Generalized Anxiety Disorder, and Alcohol related disorder deploying a waitlist control and one planned study for body dysmorphic disorder (55), but none of these products have been FDA-cleared to date.

#### Treatment as usual RCT

TAU was chosen as the comparator in three studies. TAU was the main comparator arm in a 3-arm RCT of iCBT for Irritable Bowel Syndrome (IBS), the data from which was used to support the FDA clearance of Parallel (10). Regarding the control choice, the authors Everitt et al. (47) note that “blinding is not possible for psychotherapy studies” In addition to the active intervention and TAU conditions, the study included an active comparator group, which received weekly telephone-delivered CBT sessions (compared to the active intervention group, which received only minimal telephone-delivered therapist support in addition to the iCBT program). The primary outcome was a comparison between the active intervention and the TAU comparator group. While this comparison does not control for effects related to expectation of benefit, the design does allow comparing CBT for IBS across different forms of delivery. Similarly, two DTx that received FDA clearance as SaMD for the adjunctive treatment of opioid use and substance use disorder (8, 46) used TAU or reduced TAU as comparators (see Table 1 for a description of the reduced TAU versus full TAU condition) (8).

#### Sham control RCT

About half of the reviewed studies deployed a form of sham control. It is notable that different terms were used by the authors, from digital control, placebo, sham, and attention-matched placebo control reflect the different frames of reference for evidence generation – from regulatory to pharmaceutical or psychotherapeutic trials - discussed in the introduction.

Different approaches were used to design sham content: Sham controls either replaced core treatment activities with “fake, but plausible therapies” (33) or they delivered general, disease agnostic wellbeing tips (32, 56). Another strategy was to “disarm” certain content, which means removing key aspects hypothesized to drive efficacy. For example, psychoeducational content included in the DTx may be retained in the sham control, but related quizzes or concrete skill training based on the educational content were specific to the therapeutic (32). Finally, certain content and/or features were removed in the control condition, mainly disease specific therapeutic content (e.g. CBT (32, 56)) or additional disease management features (community features (33)).

It is interesting to note that by analyzing which aspects of digital interventions were changed or removed in a digital control, we can draw conclusions about which features were considered to have the highest likelihood for being efficacious in the DTx. For example, in a trial examining SHUTi, a DTx for insomnia, the researchers did not include a sleep window suggestion in their “attention control” (56). Therefore the investigational treatment and control condition differ on at least these two aspects, and thus the difference between them might be based on core CBT content, the concrete sleep time suggestions, or both (56). Similarly, Sleepio, another DTx for the treatment of insomnia, created a digital control app that contained no social community feature compared to the active intervention (33). The authors described the social community features as mainly targeting engagement, but don't include it in the control, highlighting the fact that appropriate engagement, i.e., ensuring the patients use the DTx and experience other treatment components is relevant for treatment efficacy. The above examples highlight the complexity of designing control conditions for DTx as well as the challenge of designing inactive control conditions while keeping enough credible content for participants blinding.

### Control conditions for DTx delivered cognitive interventions

It is noteworthy that all reviewed DTx containing gamified cognitive training or remediation interventions chose a form of sham control, with some sham controls looking similar to consumer grade apps or video games. For example, Endeavor, a video game-based treatment to improve cognition in children with ADHD was compared to a digital control “word game” (letter-connecting to spell words) designed to not engage cognitive targets associated with the primary outcome (27). The study found that the treatment separated from the control on the primary, objective measure of attention, but not on clinical measures related to ADHD subjective symptoms (27). A similar example comes from Posit Science that compared their gamified cognitive training DTx targeting specific impairments in patients with schizophrenia against off-the-shelf computer games (e.g., solitaire, checkers) in a registration trial. The authors stated that the controls were “matched to the experimental treatment program in intensity and duration”, while “plausibly engaging cognitive systems” (34). While the trial was designed as a superiority trial, the authors use the term “active control” for the off-the-shelf games. The use of active control here is likely applying a more academic nomenclature of active controls which is not in line with the FDA definition where active controls are interventions with known therapeutic effects (42). In this trial, the treatment did not statistically separate on the primary outcome of cognitive functioning, but surprisingly the authors did not discuss whether their “active control” could indeed have improved cognitive functioning and may have been better described as a lower dose cognitive training (akin to lower dose control conditions in pharmacological trials). The above examples of game-based digital cognitive treatment studies point to the fact that regular, focused engagement using games as controls may not be truly inert controls as they can produce therapeutic effects.

### Blinding

Surprisingly, none of the trials report blinding checks, which are recommended by trial reporting standards (Consolidated Standards Of Reporting Trials, CONSORT (57)). This may be related to many trials being blind-to-hypothesis instead of blind-to-assignment. Nevertheless, study conditions could be compared regarding the respective expectation of benefit before a trial (see Akili's Endeavor trial for an example of expectation matching (27)) or perceived credibility of the intervention after the trial. In general, low rates of reported blinding checks are a known issue in nonpharmacologic treatments which DTx (58).

One trial for a DTx targeting schizophrenia symptoms deployed a minimal control condition during a Phase 2 study that consisted of an app displaying a count-down timer to indicate the remaining study duration (59). The study protocol offers limited information on the exact instruction for the control condition or patient's perception of being allocated to this minimal digital engagement control, it is notable that the digital control improved some of the secondary outcomes and did not perform significantly worse than the investigational treatment (59). This effect may be due to at least partially ineffective blinding in the group receiving the minimal control condition or they could be related to regular engagement with a structured digital experience, which resembles specific components discussed in psychotherapy, such as behavioral activation, discussed above. But without data on the patient experience in such a minimal, potentially unblinded digital control condition these remain hypotheses.

## Discussion

### Summary of the findings and challenges

This review highlights some key challenges in the design and description of control choices for DTx RCTs. Overall, the reviewed studies show a range of control strategies ranging from unblinded waitlist and TAU designs, to placebo-controlled trials which replace hypothesized core treatment components and often additional disease management and engagement related tools.

Control condition nomenclature across studies was inconsistent, ranging from sham or placebo control to digital control, attention-matched placebo control or active control. This may relate to different definitions of these terms in the literature, for example in the behavioral research literature versus FDA guidance (see Definitions Table 1). Thus, the names used for control conditions may not accurately describe the actual control designs, which limits the comparability of results across studies and even across fields. Similarly, a clear description of the control intervention is missing in most cases, and standardization of approach to control condition descriptions will be critical moving forward (for example following a standardized framework for intervention descriptions, such as that proposed by Tidier (60)).

Authors did not regularly discuss control design choices such as why certain features were removed or disarmed. In addition, the potential efficacy of similar control conditions is described differently across studies. For example, a wellbeing program (The Health and Well-Being Program) included as a control condition for obsessive compulsive disorder was described as an “active intervention” (61), while similar “general health and well-being educational content” was called a placebo control for a RCT for diabetes mellitus, type 2 (32). This may exemplify the “known unknowns” of mechanisms and efficacy factors in DTx and how individual authors judge the efficacy of different features differently or at the very least point to inconsistencies of describing control conditions.

Lastly, the exact instructions researchers provided to participants with regard to study conditions and blinding was often unclear. It is not often described whether participants were blind-to-assignment (i.e., expecting to receive either an active treatment or a placebo), the standard for pharmacological trials, or blind-to-hypothesis (i.e., expecting to receive one of two potential treatments for their diagnosis, with debriefing after study participation). Based on results from research into the placebo effect, such trial specifications may bias RCTs in different ways (62) and affect comparability between trials.

### Recommendations for choosing and reporting control conditions in DTx

Given the breadth of DTx content and applications, it is unlikely that there will ever be a one-size-fits-all approach to control condition design. This is in line with Blease's comment that (14) placebos in RCTs should be thought of as “moving targets designed to mimic specific interventions, rather than “as a particular kind of thing”. In line with this thought, the author recommends calling placebo interventions “control interventions” (14). For control conditions that are using the same, digital format, digital control may be a meaningful term for the field. Further, based on our review and the realization that no ‘one-size-fits-all’ control intervention exists for DTx, we conclude with recommendations for choosing and describing control conditions.

#### Choosing control conditions

**Figure 1:**
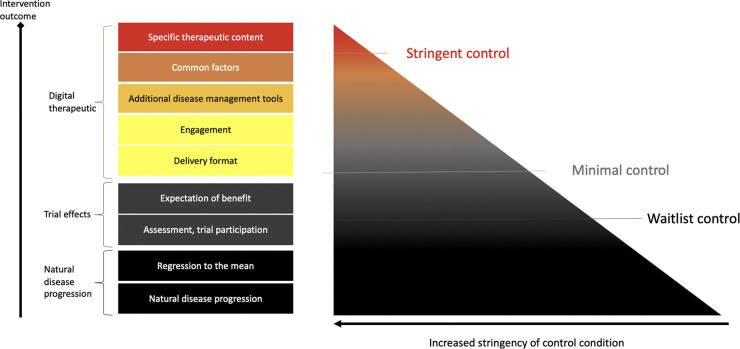
Control conditions. Control condition stringency can be distinguished with regards to what aspects are controlled for. For example, waitlist control conditions will control for aspects of natural disease progression and being regularly assessed in a trial. More stringent sham or placebo controls will also contain aspects of a digital control, such as engagement tools, digital disease management tools, such as wellness content or trackers. DTx may also try and establish a working alliance (common factors). Thus, stringent digital sham conditions may not be fully inactive.

**Figure 2:**
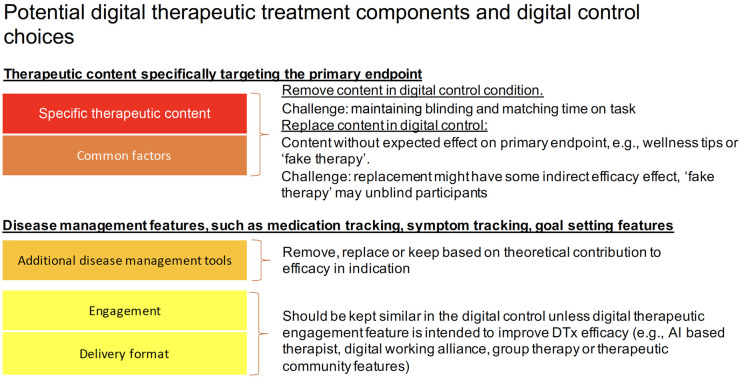
Potential choices in designing a digital control condition for DTx.

• Define the appropriate degree of stringency for digital control conditions:
  The necessary stringency of digital control conditions should reflect the risk profile and novelty of the DTx and whether it is designed to treat versus manage or prevent disease. For low-risk devices/indications that are managing versus treating diseases, less stringent control conditions may be suitable (see Figure 1 for a schematic representation of control condition choices and corresponding stringency). In addition, relatively more novel interventions may require more stringent controls compared to digital translations of known psychosocial behavioral interventions, such as CBT (12).
• Minimal control level:
  All digital control conditions should, at the very least, control for incidental effects of being in a trial (e.g., Hawthorne effect), or bias related to being assessed repeatedly (15)), disease progression over time, and regression to the mean effects. This applies to drug trials as much as to DTx. In DTx, control condition design should take into consideration additional instructions on how to use the application and time with the application should be matched as closely as possible in the digital control. We recommend that in studies which opt for a minimal control condition, data on actual engagement with the control should be part of the primary publication describing the RCT results.
• Inactive digital control conditions: To truly design an inactive digital control condition, DTx features and components designed with the intent to deliver disease specific therapeutic content should not be part of the control condition. In addition, lifestyle and disease management features (e.g., psychoeducation, pharmacological tracking, chatbots and interactions around digital working alliance, a form of working alliance that is effective in face-to-face therapy and that is actively investigated to enhance DTx engagement and efficacy (63)), motivational interviewing or features related to motivated and regular engagement should be carefully assessed as to whether they share aspects with known treatment activities and affect the primary outcome. This may include a careful literature review of known efficacy features and activities in face to face and digital interventions in a specific indication. The results of such a review and rationale for control design choices should be stated in the trial publication. At the same time, if current knowledge is limited, feasibility testing of controls may be a way to de-risk control choices before a larger RCT is conducted. The potential choices in designing a digital control condition for DTx are exemplified in Figure 2.
• Three-arm studies: Adding a third arm (e.g., TAU or Waitlist), could be helpful to add to a RCT when it is likely that a digital control condition may not be fully inert, as seen in the trial by Mahana Therapeutics (47). This will help elucidate real-world effectiveness of DTx while also providing the potential to compare to a more stringent or even active control condition.

#### Providing details on control conditions and other relevant trial information:

• Describing digital control conditions in detail: Given the active research into mechanisms of action in face-to-face and DTx interventions, both the intervention and control condition should be described in detail following already established standardized frameworks (e.g. Tidier (60)). Exploratory endpoints related to potential mechanisms of digital controls may help elucidate the underlying principles and may even inform more potent digital treatment designs down the line.
• Describing additional design characteristics related to expectation setting (informed consent) and blinding: If blinding to study condition was attempted for participants, it should be backed up with expectancy or blinding-check data in the published RCT. Further, authors should clearly state how expectations around study allocation were presented to participants. Whether participants expected two potentially active treatments versus a treatment and placebo control can influence the size of nonspecific effects of the two arms and may affect the number needed to enroll to show a significant difference between the conditions.

### Limitations

This review is based on DTx deploying cognitive, behavioral and psychosocial interventions that that fulfill the DTx definition, may have attempted to or are attempting FDA clearance, and are presented by the DTx alliance and similar trials based on the authors' awareness of the field. The review therefore does not constitute a systematic review and may not contain all control condition choices in the field. Nevertheless, by reviewing exemplary SaMD phase 2 and registration trials, the authors believe that the review represents a meaningful basis to foster discussion of a current key challenges in the field of DTx. Future systematic reviews should further elucidate DTx trial practices and provide guidance to the nascent field.

Further, the regulatory considerations provided and majority of trials in this review are US-centered, and therefore our assessment does not represent or discuss potential differences in regulatory practices beyond the US. Finally, while DTx risk profiles will in most cases be lower compared to pharmacological interventions, there are still risks to be considered and studied in DTx, ranging from technical risks, to a risk of a non-adequate treatment selection and the related risk of more general loss of confidence in treatments, or the risks of a DTx failing to detect serious symptoms such as suicidality. Future DTx clinical trials should adequately test these aspects.

### Final remarks and conclusions

RCTs remain a key aspect in evidence generation in DTx, especially those therapeutics seeking regulatory approval. However, the stringency of digital control conditions may vary based on factors such as risk profile and novelty of the intervention. Given the general low risk profile and potential of DTx to increase access to personalized care, many DTx may choose a less stringent minimal control or even waitlist control. Alternatively, minimal digital controls in the form of engagement with regular, general digital content (digital diversions) may be appropriate for efficacy studies conducted under controlled, artificial settings.

While waitlist controls may overestimate treatment effects, they may be a viable option as a control condition in large-scale real-world studies. Indeed, in light of the iterative nature of software development, there has been a call for more innovative real-world data approaches to evidence generation for DTx beyond classical RCTs (64). DTx are uniquely positioned to collect real-world data, engagement patterns, user reported outcome data, and/or clinically relevant digital phenotypes directly through their software application to assess their real-world engagement and effectiveness. Indeed, real-world and pragmatic study approaches are gaining popularity, and have been recommended to support regulatory decisions (e.g. The 21st Century Cures Act) (65).
