# Supplementary material for: Appropriate controls for digital therapeutic clinical trials: A narrative review of control conditions in clinical trials of digital therapeutics (DTx) deploying psychosocial, cognitive, or behavioral content
Source: Front Digit Health. 2022 Aug 18;4:823977. doi: 10.3389/fdgth.2022.823977 (PMC9436387; doi:10.3389/fdgth.2022.823977)
Supplement: Supplementary file 1 [file Table_1_v1.docx]

Supplementary Material

# Supplementary Data

# Table 1 Overview of reviewed DTx RCTs

| **Product (Study name)/Company/Source** | **Indication, n** | **Trial Design, Arms** | **Blinding info/Blinding check (participants, assessors, statisticians)** | **Arms (author's name for control)** | **Content** | **Duration** | **Delivery context** | **Engagement support** | **Differences in the interventions/arms** | **Primary Outcome** |  |
| --- | --- | --- | --- | --- | --- | --- | --- | --- | --- | --- | --- |
|  |  |  |  |  |  |  |  |  |  |  |  |
| Somryst (SHUTi) | Insomnia, n= 1149 | RCT, 2 arms | Double (assessors, statisticians, chief PI). *Participants were asked which of the two conditions they preferred:“Monitoring sleep activity and then doing online trainings to change sleeproutine” or “Monitoring  sleepactivity, being provided with health information, and being asked your views about the possible causes of sleepproblems” | SHUTi, CBT for insomnia | Modular insomnia program based on CBT for insomnia, daily sleep diary, recommended sleepwindow/bedtime, personalized goal setting, symptom checklist, homework assignment, quizzes | 6 weeks | internet-delivered | Email contact regimen was used to | Different treatment content. In addition, the control: did not provide sleep window suggestions, symptom checklist and goalsetting. Unclear if both continued a sleep diary after randomization. | SHUTi>Healthwatch on primary (depression symptoms PHQ-9) at 6 weeks and 6 months |  |
| Pear therapeutics |  |  |  |  |  |  |  |  |  |  |  |
| Christensen et al. 2016, ﻿ACTRN12611000121965 |  |  |  | HealthWatch (attention-matched placebo control) | Interactive lifestyle website with no specific mental health or sleep-related content (information on environmental health, nutrition myths, heart health, activity, medication, the effects of temperature, oral health, blood pressure and cholesterol, calcium, and back pain, as well as surveys about each of these topics each week) | 6 weeks | internet-delivered | treatment program clinicians were asked to incorporate brief discussion of module completion into individual counseling sessions |  |  |  |
| Sleepio | Insomnia, n=164 | RCT, 3 arms | Participants blinded to group, no blinding check reported | CBT for insomnia | CBT, including sleep information/education, relaxation, mindfulness, and social community of users moderated by experts, quizzes | 6 sessions, 6 weeks | internet-delivered, animated therapist, social community and expert moderator | Support prompts, reminders email/sms, progress reinforcement, community feature | Control: CBT content removed, social community feature removed, relaxation instruction or behavioral advice removed, | CBT>TAU and CBT>IRT on ﻿sleep efficiency at 6 weeks |  |
| Big Health |  |  |  |  |  |  |  |  |  |  |  |
| Espie et al., 2012 |  |  |  | IRT (Placebo) *(IRT was based on a well-established and credible non-pharmacological placebo intervention, contains no known active therapeutic ingredient and was used in several clinical trials) | IRT pseudo sleep training, real sleep information/education, breathing control, no quizzes, no social community | 6 sessions, 6 weeks | internet-delivered, animated therapist | Support prompts, reminders email/sms, progress reinforcement |  |  |  |
| ISRCTN - 44615689 |  |  |  |  |  |  |  |  |  |  |  |
|  |  |  |  | TAU | Diary keeping and email support only (no restrictions on usual care) | N/A | N/A | N/A |  |  |  |
| Parallel (WBCT) | Irritable Bowel Syndrome (IBS), n=558 | RCT, 3 arms, Phase 3 | Single (outcomes assessor and statistician), no blinding of participant possible | Web-based CBT (WCBT) with minimal therapist support | CBT for IBS (+TAU) | 8 online sessions/week, 9 weeks, additional homework tasks and 3x30 min telephone therapy | internet-delivered and short telephone therapy support | None described | Active control arm with lower intensity and different delivery context (phone versus web); TAU used for direct statistical comparison | WCBT>TAU in IBS Symptom Severity Score and Work and Social Adjustment Scale (WSAS) at 12 months |  |
| Mahana |  |  |  | Telephone-delivered CBT (TCBT) | CBT for IBS (+TAU) | 6x60min telephone therapy, 9 weeks, additional homework tasks | internet-delivered | None described |  |  |  |
| Everitt et al., 2015/2019 |  |  |  | TAU | TAU | N/A | N/A | N/A |  |  |  |
| reSET-O (TES) | Opioid use disorder, n=170 | RCT, 2 arms | No blinding of participants or research staff | TAU + TES | TES consists of 62 computerized interactive modules covering skills for abstinence, plus prize-based motivational incentives | 12 weeks | internet-delivered intervention (computers on site at clinics in the study) | Treatment program clinicians were asked to incorporate brief discussion of module completion into individual counseling sessions | No digiital intevention present in the TAU group used for statistical comparison | TES > TAU in total half weeks of opioid abstinence and less dropout from treatment |  |
| Pear therapeutics |  |  |  | TAU (in person Buprenophine administration 3xweeks, clinician visit every other week) | TAU consisted of individual and group counseling at the participating programs | 12 weeks | internet-delivered | N/A |  |  |  |
| Christensen et al. 2014 |  |  |  |  |  |  |  |  |  |  |  |
| reSET (TES) | Substance use disorder, n=507 | RCT, 2 arms | No blinding of participants or research staff | Reset + reduced TAU | Reduced TAU (only 2 hours weekly sessions) | 12 weeks | internet-delivered |  |  | TES > TAU in abstinence rate and less dropout from treatment |  |
| Pear therapeutics |  |  |  |  |  |  |  |  |  |  |  |
| Campbell et al., 2014 |  |  |  | TAU | Group or individual therapy sessions at least twice a week, 4-6 hours | 12 weeks | internet-delivered |  |  |  |  |
| Endeavor (AKL-T01) | Pediatric ADHD, n=348 | RCT, 2 arms | Tripple (participants blinded for hypothesis not for treatment) | AKL-T01 | Multitasking training | 25min/day, 5 days/week, 4 weeks | digital, iPad app | App reminders, trial staff reminders | Different core intervention, same time on task, progression and engagement support | AKL-T01>Digital control on objective measure of attention |  |
| Akili Interactive |  |  |  |  |  |  |  |  |  |  |  |
| Kollins et al., 2020 |  |  |  | Digital control intervention | Digital word search game | 25min/day, 5 days/week, 4 weeks | digital, iPad app | App reminders, trial staff reminders |  |  |  |
| Posit Science | Schizophrenia, n=150 | RCT, 2 arms (designed to be a pivotal trial for this spe- cific implementation of computerized brain-plasticity-based cogni- tive training in schizophrenia) | Participants, neuropsychological testers, and clinician raters were blinded (participants blinded for hypothesis not for treatment) | ﻿Computerized cognitive training in a game-like format | Custom-built for people with schizophrenia, 16 cognitive exercises delivered as part of an overarching game wrapper. All exercises targeted speed and accuracy of information processing | 1h/day, 5 days/week, 26 weeks | internet-delivered | In-clinic visits for the training. Coach available on request. In game: ﻿participants earned virtual cash fromcompleting trials in the cognitive exercises, and at the end of each day's training session, the participant could spend their virtual cash in a virtual store to decorate, customize, and expand their virtual apartment. | ﻿Both the experimental treatment and the active control were software-implemented programs, designed to be self-administered by a participant using a personal computer connected to the internet. Participants trained in clinic, typically in a room with several computers, and a coach available to answer questions or provide technical help. | ﻿No significant effect treatment > active control on primary or secondary outcome measures (cognitive batteries) |  |
| Mahncke et al. (2019) |  |  |  |  |  |  |  |  |  |  |  |
|  |  |  |  | Computer games (Active control) | ﻿The active control programwas designed to provide an experience that could be matched to the experimental treatment program in intensity and duration, while plausibly engaging cognitive systems to maintain the patient blind. Thirteen off-the-shelf computer games were selected (e.g., Solitaire, Checkers), and deliveredwith a schedule similar to the experimental treatment. | 1h/day, 5 days/week, 26 weeks | internet-delivered |  |  |  |  |
| PEAR-004 | Schizophrenia, n=112 | RCT, Phase 2, 2 arms | Single (outcomes assessor), participant blinding unclear | Clinician-directed pharmacotherapy + PEAR-004 | Suggestions about coping strategies to overcome difficulties in daily life | 12 weeks | smartphone app | None described | Control: did not contain any real or pseudo treatment content. | No significant difference |  |
| Pear therapeutics |  |  |  |  |  |  |  |  |  |  |  |
| ClinicalTrials.gov: NCT03751280 |  |  |  | Clinician-directed pharmacotherapy + sham app (Sham) | Sham control app: prescription timer (duration of app availability) | 12 weeks | smartphone app | Receive notifications prompting the participant to open the sham app |  |  |  |
| Perspectives OCD | Obsessive compulsive disorder (OCD), n=120 | RCT, 2 arms | Single (Outcomes Assessor) | CBT for OCD | The app-delivered CBT in this project includes modules such as cognitive skills (e.g., cognitive restructuring, core belief work) and behavioral skills (e.g., exposure and response prevention). | 12 weeks | smartphone app | In-app coaching function to facilitate low-effort encouragement and accountability when people need it most. |  | Not completed, primary endpoint: Difference in OCD severity |  |
| KAO Health |  |  |  |  |  |  |  |  |  |  |  |
|  |  |  |  |  |  |  |  |  |  |  |  |
| NCT04136626 |  |  |  | The Health and Well-Being Program (Active Comparator) | The website includes 12 modules related to general health and well-being (e.g., sleep and nutrition). Each module contains educational information and questions about an indvidual's experience with the given topic. | 12 weeks | internet-delivered | Unclear |  |  |  |
| KOA Health for BDD | Body Dysmorphic Disorder (BDD), n=80 | RCT, 2 arms | Single (Outcomes Assessor) | CBT for OCD | cognitive skills (e.g., cognitive restructuring, core belief work), behavioral skills (e.g., exposure with ritual prevention), and perceptual retraining/mindfulness skills. | 12 weeks | smartphone app | In-app coaching function to facilitate low-effort encouragement and accountability when people need it most. | Expectation of benefit not controlled | Not completed, primary endpoint: Difference in BDD severity |  |
|  |  |  |  |  |  |  |  |  |  |  |  |
| NCT04034693 |  |  |  | Waitlist control | participants will be crossed over to 12-week Smartphone-delivered CBT for BDD following the 12-week waitlist control) | 12 weeks | Waitlist | In-app coaching function to facilitate low-effort encouragement and accountability when people need it most. |  |  |  |
| Happify | Diabetes (Type 2), n=450 | RCT, 2 arms | Not reported, clinicaltrials mentions: None (Open Label) | Happify Health: diabetes-specific track that focuses on building skills for greater happiness, reducing stress, and coping better with diabetes. In addition: Users may access a wide variety of 4-week programs | Positive emotion regulation skills  intervention group | 8 weeks | smartphone app | Daily push notifications/reminders on their mobile device; weekly emails | Sham condition consists of access to a version of | Not completed, primary endpoint: HbA1C |  |
| Happify Health |  |  |  |  |  |  |  |  |  |  |  |
| Boucher et al., 2020 |  |  |  | Digital sham (Happify Health partial content) | Psychoeducational content': polls on wellbeing topics, provided with some social comparison data, as well as information about why well being is important | 8 weeks | smartphone app | Daily push notifications/reminders on their mobile device; weekly emails | Happify Health that includes polls on various mental health topics and other pseudoeducational content |  |  |
| Deprexis | Depression, n=376 | RCT, 2 arms | No blinding of participants or research staff | Deprexis | 10 modules including behavioral activation, mindfulness, interpersonal skills) | 8 weeks | internet-delivered | Minimal: pre-randomizaiton phone call, reminders if assessments were not completed or app was not used during a 2 week period. | Waitlist control receives no intervention. | Deprexis>WLC: Depression (QIDS-SR) |  |
| Orexo |  |  |  | Waitlist control |  | 8 weeks | NA |  |  |  |  |
| Beevers, 2017 |  |  |  |  |  |  |  |  |  |  |  |
| Daylight | Generalized Anxiety Disorder, n=256 | RCT, 2 arms | Participants and the trial coordinator were not blind to group allocation, other research personel and statistician blinded | Daylight | personalized digital CBT (applied relaxation, stimulus control, cognitive restructuring, and imaginal exposure) | 6 weeks | smartphone app | patient opted in engagement text messages, and push notifications. | Waitlist control receives no intervention. | Daylight>WL in anxiety symptoms (GAD-7) and other measures (depressive symptoms, sleep difficulty, wellbeing, and participant-specific quality of life) |  |
| Big Health |  |  |  | Waitlist control |  | 6 weeks | NA |  |  |  |  |
| Carl, 2020 |  |  |  |  |  |  |  |  |  |  |  |
| Vorvida | Alcohol related disorder, n=608 | RCT, 2 arms | No blinding of participants or assessors | Vorvida | CBT | 6 months | internet-delivered | not reported | Waitlist control receives no intervention. | Vorvida>WL in self-reported alcohol consumption |  |
| Orexo |  |  |  |  |  |  |  |  |  |  |  |
| Zill, 2019 |  |  |  | Waitlist control | NA | 6 months | NA |  |  |  |  |
